# An internet-delivered psychoeducational intervention (Fex-Can 2.0) targeting fertility-related distress and sexual dysfunction in young adults diagnosed with cancer: Study protocol of a randomized controlled trial with an internal pilot phase

**DOI:** 10.1371/journal.pone.0322368

**Published:** 2025-04-29

**Authors:** Rebecca Skog, Erik M.G. Olsson, Jessica R. Gorman, Sharon L. Bober, Claudia Lampic, Lena Wettergren

**Affiliations:** 1 Department of Public Health and Caring Sciences, Uppsala University, Uppsala, Sweden; 2 Department of Women’s and Children’s Health, Uppsala University, Uppsala, Sweden; 3 College of Health, Oregon State University, Corvallis, Oregon, United States of America; 4 Department of Supportive Oncology, Dana-Farber Cancer Institute, Boston, Massachusetts, United States of America; 5 Department of Psychiatry, Harvard Medical School, Boston, Massachusetts, United States of America; 6 Department of Psychology, Umeå University, Umeå, Sweden; 7 Department of Women’s and Children’s Health, Karolinska Institutet, Stockholm, Sweden; PLOS: Public Library of Science, UNITED KINGDOM OF GREAT BRITAIN AND NORTHERN IRELAND

## Abstract

**Background:**

While previous literature has continuously demonstrated the negative effects of cancer and its treatment on fertility and sexuality, evidence-based interventions to alleviate fertility-related distress and sexual dysfunction are lacking. This study protocol describes the internal pilot study and randomized controlled trial of an internet-delivered psychoeducational intervention: Fex-Can 2.0. The primary objective is to determine efficacy of Fex-Can 2.0 in terms of reduction of fertility-related distress and sexual dysfunction at end of the 12-week intervention. The internal pilot study will assess feasibility of the study, determined according to pre-specified progression criteria and individual interviews.

**Methods:**

The study has a randomized controlled design, with an internal pilot phase. The intervention group will receive Fex-Can 2.0, consisting of psychoeducational- and behavior change content. The control group will be allocated to standard care. Primary outcomes are fertility-related distress (RCAC) and sexual function and satisfaction (PROMIS SexFS Brief Sexual Profile). Secondary outcomes include body image (BIS), emotional distress (HADS), health-related quality of life (EORTC QLQ-C30), need satisfaction and frustration scale (NSFS), fertility- and sex-related knowledge, and self-efficacy related to fertility and sex life. Outcomes will be assessed at baseline, directly after the intervention, and 12 weeks later. During the internal pilot, data on trial recruitment, data collection, drop out, and adherence will be collected to assess feasibility. Semi-structured interviews will be conducted to further assess acceptability of Fex-Can 2.0.

**Conclusions:**

This randomized controlled trial aims to evaluate whether Fex-Can 2.0 is superior to standard care, in terms of reducing fertility-related distress and sexual dysfunction in young adults diagnosed with cancer. If proven efficacious, the Fex-Can 2.0 intervention may be a valuable resource in health care, with the potential to significantly improve the care of young adults experiencing fertility-related distress and/or sexual dysfunction following cancer.

**Trial registration:**

ClinicalTrials.gov ISRCTN18040643

## Introduction

Sexual- and reproductive health is important for the quality of life of young adult (18–39 years) cancer survivors [1,2]. As a result of cancer and its treatment, young adults commonly report fertility-related distress and sexual dysfunction [3,4]. Several of the most common cancer treatments can cause temporary or permanent sub-or infertility [5]. Individuals diagnosed with cancer during their reproductive years’ experience substantial distress about their fertility [6,7]. In a population-based study including 1010 participants, 54% of female, and 27% of male young adult cancer survivors reported high-levels of fertility-related distress approximately 1.5 years post-diagnosis [7]. Such distress covers not only actual or potential infertility, but a broad array of concerns related to fertility and parenthood [8]. In addition, cancer-related sexual problems may occur as a result of disease and treatment [9,10] and are comprised of physiological, psychological, interpersonal, and social and cultural elements [10]. A majority of young adults experience some degree of sexual dysfunction following cancer [11,12], including low sexual interest [12,13]. Female survivors commonly report vaginal dryness, pain and discomfort [12,14], and male survivors report problems related to erectile and/or ejaculatory dysfunction [15,16]. The experiences of fertility-related distress and sexual dysfunction have been associated with emotional distress [4,12], and may negatively impact partner relationships [17,18], body image [12,19], and sense of femininity or masculinity [20,21].

In Sweden, approximately 2,300 young adults are diagnosed with cancer every year [22]. As the young adult cancer population is relatively small, and those living with and beyond cancer are geographically disperse, the delivery of online interventions appears promising for increasing access to supportive care [23]. However, few internet-delivered interventions have focused on fertility-related distress and sexual dysfunction, and there is a lack of evidence-based interventions in place to alleviate such problems for young adult cancer patients. Existing fertility-related interventions have predominantly targeted female survivors, and have mainly been fertility preservation decision aids [24], or educational interventions [25,26]. Meneses et al [26] evaluated an internet-based reproductive health and fertility education program for young breast cancer survivors (18–50 years) using a pre-post study design. At post-intervention, participants had significantly improved in physical and social functioning and fertility-related knowledge [26]. Internet-delivered interventions for sexual problems following cancer have predominantly targeted males with prostate cancer [27–29], and inclusion of young adults in interventions targeting diagnoses more common in the age group (e.g., breast-, gynecological cancers) have been limited, with mean ages of participants in previous studies ranging between 39.9 to 53 years [30–32]. Interventions targeting sexual problems after cancer have included various CBT-techniques [30,32,33], psychoeducation [30,31,34], mindfulness [34], and opportunity for interaction with peers and/or health care providers [30,31,34]. Promising results have been demonstrated, for example in improving sexual satisfaction [29,32] and functioning [30,33].

A common limitation of internet-delivered interventions is low levels of adherence and high attrition rates, which may result in insufficient sample sizes and threaten the validity of findings [35]. Reasons for low adherence and discontinued use of interventions are likely to be multifaceted [35,36]. In one study on early drop-out and adherence to an internet-based CBT-program, overall study attrition was found to be associated with experiencing a low level of intrinsic motivation [37], which is a highly autonomous form of motivation [38,39]. Self-determination theory (SDT) differentiates between autonomous and controlled forms of motivation [38,39]. Autonomously motivated behaviors are performed for self-endorsed reasons, while controlled forms of motivation refers to engaging in behaviors for externally or internally pressured reasons (e.g., rewards, social approval, avoiding punishment) [39].

SDT further posits that humans have three basic psychological needs: competence (feeling effective in one’s behavior), autonomy (sense that own actions are volitional rather than controlled), and relatedness (need to connect with others) [39], and these needs are essential for well-being and optimal functioning [38,39]. When the psychological needs are supported, behaviors are likely to be perceived to be performed autonomously, which in turn, may increase the likelihood of behavior maintenance and persistence [38,39]. SDT has been utilized as a theoretical foundation in previous studies aiming to support and maintain positive changes in health behaviors and improve physical and psychological health [38,40]. Interventions that are supportive of the basic psychological needs have been found to be associated with greater internalization and behavior persistence [38,41]. In the context of sexual health following cancer, Bober et al [42,43] developed a group-based psychosexual intervention guided by SDT. The intervention aimed to improve sexual functioning following treatment for ovarian cancer, and to increase participants self-efficacy and competence to address problems experienced. At post-intervention, participants self-efficacy had improved significantly, and was further associated with increased sexual functioning [43].

The study presented in this study protocol is part of the Fertility and Sexuality following cancer (Fex-Can) project [44,45]. The Fex-Can project investigates fertility-related distress and sexual dysfunction among young adults during the first five years following a cancer diagnosis [44], and aims to develop and evaluate an internet-delivered intervention to alleviate such problems [45]. An original version of the Fex-Can intervention was developed in collaboration with patient research partners [46], and consisted of two separate programs: Fex-Can Fertility and Fex-Can Sex. Both programs were evaluated in randomized controlled trials (RCT). Participants allocated to the intervention group (IG) of the Fex-Can Fertility program reported significantly lower distress with regards to child’s health, and better self-perceived cancer-related fertility knowledge following the program, as compared to the standard care control group (CG) [47]. The Fex-Can Sex program did not yield any statistically significant improvements among the IG as compared to the CG [48]. Fex-Can 2.0, presented in this manuscript, is adapted from the original intervention, and aims to overcome identified shortcomings by refining and improving the intervention and study procedures.

### Objectives

The present study protocol describes the design of an internal pilot study and RCT to evaluate the internet-delivered psychoeducational intervention Fex-Can 2.0. The internal randomized controlled pilot trial aims to evaluate the feasibility and acceptability of the Fex-Can 2.0 intervention, in terms of trial recruitment, data collection, drop out, adherence, and experiences of the intervention. The aim of the RCT is to evaluate whether the Fex-Can 2.0 intervention is superior to standard care, in terms of reduction of fertility-related distress and sexual dysfunction in young adults diagnosed with cancer. Further, the study will assess whether the intervention has effects on secondary outcomes, including body image, health-related quality of life, emotional distress, fertility- and sex-related knowledge, and self-efficacy related to fertility and sex life. Finally, to understand how and under what circumstances potential effects of the intervention are achieved, satisfaction with basic psychological needs will be assessed in a theory-based mediation analysis.

### Trial design

The study will be performed in a two-armed superiority RCT, with a 1:1 allocation ratio. The intervention group will receive the Fex-Can 2.0 intervention, while the control group will be allocated to standard care. The first 70 participants enrolled will be included in the internal pilot study. During the internal pilot study, feasibility will be assessed against pre-specified progression criteria (described in detail under heading ‘Progression criteria’) [49]. A stop-amend-go system is adopted for evaluation of potential progression to a full-scale RCT. Semi-structured interviews with IG participants will be conducted to explore experiences of participation. The study protocol adheres to the SPIRIT statement for clinical trial protocols (S1 Spirit Checklist in S1 File) [50], as well as the template for intervention description and replication (TIDieR) checklist (S2 TIDieR Checklist in S2 File) [51]. All items from the WHO Trial Registration Data Set are included as supporting information (S3 WHO Trial Registration Data Set in S3 Table).

**Fig 1:**
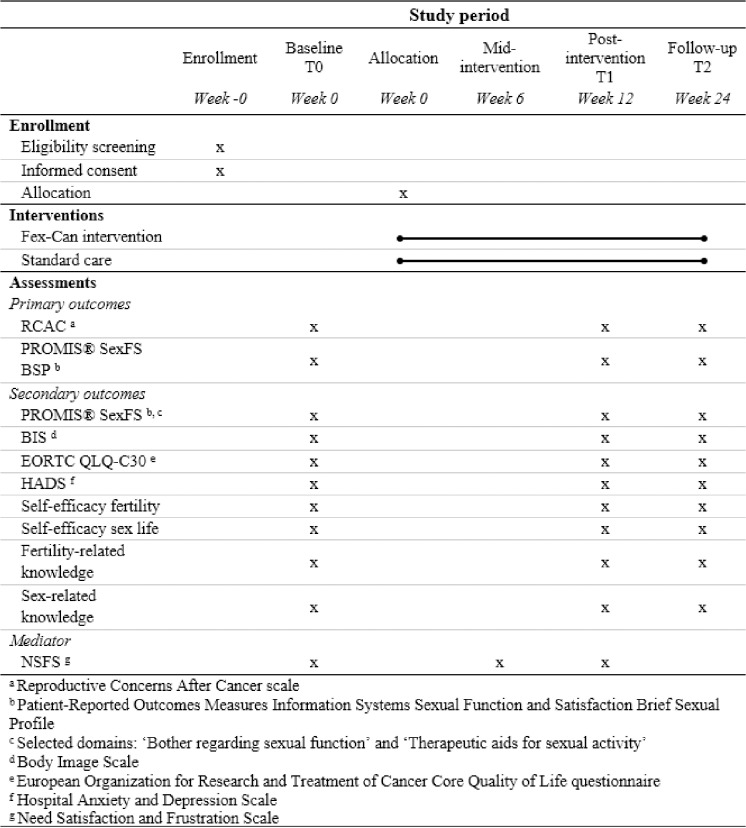
Schedule of enrolment, interventions and assessments.

**Fig 2:**
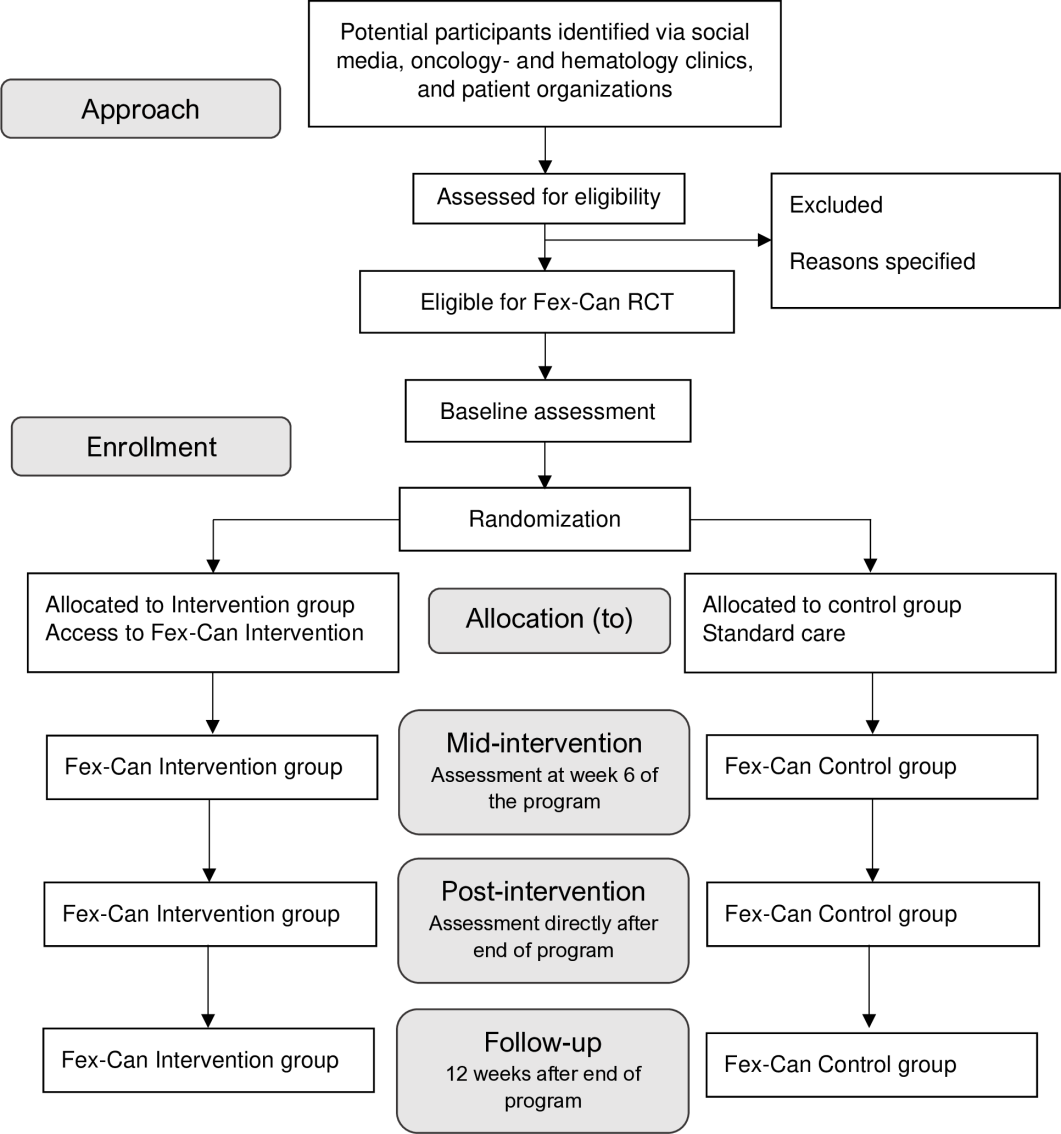
CONSORT standard RCT flow diagram.

Fig 1 presents the flow chart and schedule of enrolment, interventions and assessments of the trial. Fig 2 presents the Consolidated Standards of Reporting Trials (CONSORT) flow diagram.

## Methods

### Study setting

The study will be conducted in a fully remote setting. The intervention will be delivered on the U-CARE internet platform, which is a platform developed by the Uppsala University Psychosocial Care (U-CARE) Programme to provide a secure infrastructure for delivery and evaluation of internet-delivered interventions [52]. Study participants will be recruited via adverts on social media, through patient advocacy organizations and through oncology- and hematology clinics in Uppsala, Stockholm and Umeå, Sweden.

At submission of the study protocol, recruitment of study participants has not started, nor has any data been collected. Recruitment of study participants is planned to begin in May 2025, and the internal pilot study will start in September 2025. Results regarding feasibility are planned to be reported during 2026–2027. If feasibility of Fex-Can 2.0 is demonstrated, the study will progress into the full-scale RCT immediately, with results expected to be reported at the latest 2031.

### Eligibility criteria

Eligibility criteria will be assessed through screening interviews with a research team member. Individuals are eligible for participation in the Fex-Can 2.0 study if:

1. Diagnosed with cancer within the past 5 years
2. Aged 18–39 years at study entry
3. Experiencing significant fertility-related distress and/or sexual problems
4. Prepared to spend at least 30 minutes per week on the intervention website.

Exclusion criteria for the present study include:

1. Inability to communicate in Swedish
2. Suicidality or a significant psychiatric condition

### The Fex-Can 2.0 intervention

Fex-Can 2.0 is an internet-delivered psychoeducational guided self-help intervention, delivered in modules over a 12-week period. Prior to accessing the modules, participants will take part of an individual online start-up session, during which problems and experiences will be discussed with a research team member. Based on the start-up session, participants will be recommended an individually selected set of modules relevant to their problems and needs. After working through an introductory module (‘Fertility and sexuality follow cancer’), participants will follow their recommended modules through the program. Guidance, in form of written feedback and individual online start-up and exit sessions, will be provided by researchers with a background in psychology, nursing, physiotherapy and public health.

Fex-Can 2.0 consists of key components of internet-delivered interventions, as defined by Barak et al [53] including structured behavior change-content, multimedia, interactive online activities, and feedback support. More specifically, modules consist of psychoeducational and behavior-change content including general- and cancer specific fertility- and sexuality related information, illustrations and images, personal stories of experiences of fertility and sexuality as told by young adult cancer survivors, self-monitoring exercises and interactive components (e.g., quizzes and reflective questions), and exercises inspired by mindfulness and cognitive behavioral therapy (CBT) with personal and automated written feedback. Throughout the intervention period, participants will have access to a moderated asynchronous peer discussion forum. The adaption and refinement of the original Fex-Can intervention was done in collaboration with a group of patient research partners, consisting of young adults with a cancer experience. Table 1 provides an overview of the intervention structure and included modules.

**Table 1:** Description of the Fex-Can 2.0 intervention.

General features	
Platform	Uppsala University Psychosocial Care (U-CARE) Program Platform
Start-up session	Mandatory online session to describe problems and experiences. Based on this, relevant modules will be recommended to participants.
Exit session	Optional online session to discuss participants experiences of the intervention
**Modules**	
Fertility and sexuality following cancer	Handling emotional distress
Relationships	Trying to have children following cancer
Own and child’s health	My body
Orgasm (females)	Discomfort and pain (females)
Erection (males)	Orgasm and ejaculation (males)
Not able to have biological children	Sexual desire
**Content**	
Content of modules	Psychoeducational- and behavior change content: informational texts, multimedia, interactive online activities, videos and texts by young cancer survivors.
Quizzes	Testing knowledge of fertility- and sexuality-related topics presented in the modules, to facilitate engagement and learning
Reflective questions	Example: “What does sexuality mean to you and what are your expectations for the program?”
Exercises	Examples: mindfulness, pelvic floor exercises, exploring physical touch (alone/with sex partner)
Discussion forum	Moderated and asynchronized, using pseudonymized aliases

#### Theoretical framework.

**Fig 3:**
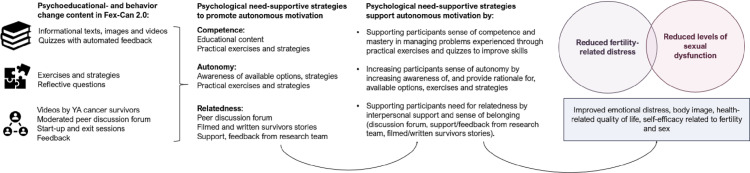
Program theory Fex-Can 2.0.

The content and delivery of Fex-Can 2.0 is guided by SDT, and addresses the basic psychological needs that are essential for well-being, and for facilitating autonomous motivation [38,39]. The educational- and behavior change content included is developed to support participant’s (i) capacity to take actions to improve or change their situation (competence), (ii) sense of being in control over their lives and actions (autonomy), and (iii) sense of belonging (relatedness). In line with previous literature, participants whose basic psychological needs are supported through the intervention activities are hypothesized to feel autonomously motivated to engage with the content, and in the long-term, experience greater impact on their fertility-related distress and/or sexual dysfunction [38,54]. As described by Pingree et al [54], and further demonstrated by Hawkins et al [55] and Hull et al [56], we hypothesize that effects of the intervention may be mediated by satisfaction of these three basic needs. The program theory for Fex-Can 2.0 is presented in Fig 3.

### Control condition

The outcomes of the intervention group will be compared to that of a control group allocated to standard care. According to the Swedish National Clinical Cancer Care Guideline for cancer rehabilitation [57], health care professionals are recommended to inform patients about the potential impact of cancer and cancer treatment on sexual and reproductive health, and to assess needs for rehabilitation. However, previous studies have demonstrated that not all patients receive sexual and reproductive health information from their health care providers [58,59]. Thus, standard care may or may not include fertility- and/or sexuality-related support and scheduled contacts with health care depending on type of diagnosis and clinic. As no other alternative treatment is established, standard care was selected as a comparator.

### Adherence

Participants’ adherence to the intervention will be evaluated through log in and activity data from the platform, including number of log ins, number of modules completed, number of exercises completed, and posts made in the discussion forum. Additionally, interviews and items included in the post-intervention survey will address the frequency of use of various intervention functions.

Various measures will be taken to support adherence to the intervention. To be eligible for study participation, participants must be prepared to spend at least 30 minutes/week on the intervention website. At opening of each new module, participants will be notified via text message and/or e-mail. Reminders will further be sent out to participants that are inactive more than a week.

### Concomitant care

Concomitant care for fertility-related distress and sexual dysfunction will be allowed. At all assessments, participants will be requested to respond to questions about whether they have sought out care for fertility-distress and/or sexual problems.

### Study measurements

Measurements in the present study includes instruments as well as study-specific measures. Instruments will be used and scored per their respective manuals. See Fig 1 for a time-line for the administration of the study instruments.

### Primary outcomes

#### Fertility-related distress.

Fertility-related distress will be assessed using the summary score of the multidimensional Reproductive Concerns After Cancer scale (RCAC) [8,60]. The RCAC was developed for use in young adult female cancer survivors [8], and has since been adapted for use among male cancer survivors [61]. The RCAC consists of six three-item subscales: Fertility potential, Partner disclosure, Child’s health, Personal health, Acceptance, and Becoming pregnant (female version)/ Achieving pregnancy (male version). Responses are given on a 5-point scale, and refer to participants’ current thoughts and feelings about their fertility at time of responding. Higher scores reflect higher levels of concerns. The RCAC has been translated and culturally adapted, and subsequently psychometrically evaluated for use in the Swedish setting [62]. The scale has demonstrated construct and known-groups validity, and satisfactory reliability [60,62].

#### Sexual function and satisfaction.

Sexual function and satisfaction will be assessed using the PROMIS Sexual Function and Satisfaction measure version 2.0 Brief Profile (PROMIS® SexFS BSP v2.0) [63]. The BSP female version consist of 13 items from 8 domains: Interest in sexual activity, Vaginal lubrication, Vaginal discomfort, Vulvar discomfort labial, Vulvar discomfort clitoral, Orgasm ability, Orgasm pleasure and Satisfaction with sex life. The male version consists of 9 items from 5 domains: Interest in sexual activity, Erectile function, Orgasm ability, Orgasm pleasure, and Satisfaction with sex life. Both profiled further include 2 sexual activity screeners. The items refer to participants’ experiences during the past 30 days.

Domain scores will be calculated using item response theory. Domain scores will be transformed into a T-score metric, where 50 represents the mean for the American general population (SD=10). Sexual dysfunction is defined as 1 SD (10 points on the T-scale) from the population mean of 50. Participants who report not having had any individual or partner sex in the past 30 days answer only the interest domain. In line with a study by Schover et al [33], a summary BSP T-score will be used as primary outcome. The summary score will be created by averaging T-scores with equivalent weights across the domains included in the Brief Sexual Profiles (female/male versions). A BSP change score will be created by subtracting each participant’s baseline summary BSP score from the BSP score at follow-ups. The SexFS v2.0 has shown adequate content, construct and known-group validity, and test-retest reliability [63,64]. The items of the BSP have previously been translated into Swedish and culturally adapted in accordance with FACITrans and PROMIS [65,66].

### Secondary outcomes

#### Sexual function and satisfaction.

Additional items of the PROMIS® SexFS v2.0 [63] will be included to assess use of therapeutic aids for sexual activity and the degree of bother experienced with different aspects of sexual functioning.

#### Body image.

The summary score of the Body Image Scale (BIS) will be used [67] to assess body image disturbance. BIS is a 10-item scale developed for use in cancer populations, and includes affective-, behavioral-, and cognitive body image items. Responses refer back to how the respondent has felt during the past week. The scale has demonstrated reliability, clinical validity, and sensitivity to detect change over time [67].

#### Health-related quality of life.

Health-related quality of life will be assessed using the summary score of the EORTC QLQ-C30 (version 3.0) [68,69]. The questionnaire was developed for use in clinical trials and consists of five functional scales (physical, role, cognitive, emotional, and social), three symptom scales (fatigue, pain, and nausea and vomiting), a global health status scale, and six single items. The summary score of the EORTC QLQ-C30 has shown to be a reliable and valid measure of health-related quality of life in cancer patients [68,69].

#### Emotional distress.

The Hospital Anxiety and Depression scale (HADS) will be used to assess anxiety and depression [70]. The summary scores of the two 7-item subscales will be used to assess symptoms of anxiety and depression, respectively. Items refer back to how the respondent has felt during the past week. The HADS has shown satisfactory internal consistency and validity [71,72].

#### Self-efficacy related to fertility and sex life.

Change in self-efficacy related to fertile ability and sex life will be assessed by two separate, study-specific questionnaires consisting of 5 and 6 items, respectively [45]. The questionnaires assess respondents’ confidence in own ability to deal with situations, thoughts, and emotions related to fertility and sexuality. The questionnaires include statements such as “I feel confident that I can manage negative thoughts and feelings about my ability to have children/my sex life”. Responses are given on a 4-point Likert scale, and reflect experiences during the past 30 days. Mean scores are calculated, with higher scores indicating higher levels of self-efficacy related to fertility and sex life, respectively.

#### Self-reported fertility- and sex-related knowledge.

Self-reported level of knowledge of fertility and sex will be assessed using two separate study specific questionnaires, consisting of 9 and 10 items, respectively. The questionnaires are divided into two domains: general fertility-related knowledge (4 items) and cancer-related fertility-knowledge (5 items), and general sex-related knowledge (5 items) and cancer-related sex-knowledge (5 items). General fertility/sex-related knowledge cover items such as “I have good knowledge regarding the importance of age for the ability to have children“ and “I have good knowledge regarding what happens in the body during sexual arousal“. Cancer-related fertility/sex knowledge includes items “I have good knowledge regarding the effect of cancer and cancer treatment on reproductive ability“ and “I have good knowledge about how cancer treatment can impact sexuality“. Responses are recorded on a four-point scale, with higher mean scores indicate higher levels of perceived fertility and sex-related knowledge, respectively.

### Mediator

#### Satisfaction and frustration with basic psychological needs.

To assess whether effects of the intervention are mediated through the basic psychological needs, the Swedish version of the Need Satisfaction and Frustration Scale (NSFS) will be used [73,74]. The NSFS assesses need satisfaction as a source of motivation, in line with SDT [41]. The NSFS comprises 18 items, with six items for each basic psychological need (autonomy, competence and relatedness). For each need, there are three items that assess need satisfaction and three that assess need frustration. Items have been adapted to fit the context of the study. As suggested by Aurell et al [75], responses are given on a 5-point Likert scale ranging from ‘Very seldom or never’ to ‘Very often or always’. Scores will by calculated by reversing the frustration items of each scale and summarizing it with the satisfaction items. Higher scores indicate higher level of need satisfaction in each psychological need, respectively. The Swedish NSFS has been validated in the Swedish general population [74].

### Sociodemographic and clinical data

Sociodemographic and clinical characteristics will be collected through self-reports. Sociodemographic variables include gender identity, age, country of birth, level of education, sexual orientation and living situation. Clinical variables will include data on type and year of diagnosis, prior and current cancer treatment, and potential comorbidities. Additionally, data will be collected on reproductive aspects (e.g., use of fertility preservation, desire for children, fertility problems before/after diagnosis), sexual aspects (e.g., satisfaction with sex life pre-diagnosis), and on whether participants have received information about potential impact of their cancer treatment on fertile ability and sex life.

### Participant time-line

Eligible participants returning online written informed consent and the baseline questionnaire (T0) are included in the study and will subsequently be randomized to either intervention- or control group. Following randomization, participants in the IG will be invited to an individual start-up session with a research team member, during which modules will be recommended based on the individual participants problems and needs. The IG will subsequently receive access to their recommended modules delivered on the U-CARE platform over a period of 12 weeks. Half-way through the intervention (week 6), participants will be requested to respond to the NSFS. All participants will be requested to respond to surveys either online (via the U-CARE platform) or on paper directly at end of the intervention and 12 weeks later. Participants will receive two cinema tickets (valued at approximately 20 Euros) per completion of each assessment.

### Program evaluation

#### Post-intervention survey.

As part of the post-intervention assessment (T1), IG participants will further respond to questions covering usage and perceptions of intervention components. Responses are given on a four-point scale ranging from ‘Disagree completely’ to ‘Completely agree’. Further, participants will be asked to rate to what extent their fertility-related distress and/or sexual problems have changed as compared to before the intervention. Responses are recorded on a 7-point scale, with alternatives ranging from ‘Much improved’ to ‘Much worsened’.

### Evaluation of the internal pilot study

#### Progression criteria.

The trial will be evaluated according to pre-specified progression criteria (Table 2), developed based on Avery et al [49]. A stop-amend-go system will be utilized, meaning that results of the internal pilot phase will indicate whether the trial can progress to a full-scale RCT immediately, progress following amendments, or whether not to proceed. If criteria are met to a satisfying degree, and the study subsequently progresses into the full-scale RCT, data from the pilot will be included in the final analyses [49,76]. The progression criteria were set considering the possibility of finalizing recruitment in approximately 1.5 years, recruiting participants with significant levels of fertility-related distress and/or sexual dysfunction, having enough participants remaining in the study until study completion and completing assessments, as well as having a sufficient activity level to draw conclusions about use of the intervention.

**Table 2:** Pre-specified progression criteria of the Fex-Can 2.0 internal pilot trial.

	Go ^a^	Amend ^b^	Stop ^c^
**Recruitment and enrolment**			
Time needed to recruit 70 participants	≤6 months	7-8 months	>8 months
Proportion of randomized participants scoring either ≥1 SD from the population mean of the PROMIS® SexFS BSP ^d^ or ≥4 in at least one subscale of the RCAC ^e^ at the baseline assessment	≥75%	50-74%	<50%
**Drop out**			
Rate of randomized participants dropping out of the study ^f^	≤10%	21-30%	>30%
**Adherence**			
Proportion of intervention group participants reaching the level of intended usage ^g^	≥60%	40-59%	>40%
**Intervention delivery**			
Time needed for team members to deliver start-up and exit sessions, provide feedback and technical support ^h^			
Time needed for reminder contact at different time points ^h^			
**Participants experiences of the intervention**			
Intervention group participants experiences of the intervention ^h^			

Note: Progression criteria are set to be analyzed separately.

^a^No concerns regarding feasibility and/or acceptance, proceed with RCT without amendments.

^b^Potential concerns regarding feasibility and/or acceptance, minor amendments may be necessary before proceeding with RCT.

^c^Indication of low feasibility and/or acceptance, do not proceed with RCT unless changes are possible.

^d^Patient-reported outcome measures Sexual Function and Satisfaction measure.

^e^Reproductive Concerns After Cancer scale.

^f^Drop out is defined as actively leaving the study and/or not completing the post-intervention assessment.

^g^Completing at least 75% of allocated modules, including exercises, quizzes and reflecting questions.

^h^Judged feasible by qualitative data.

#### Semi-structured interviews.

To explore experiences of the intervention and study procedures, a subset of internal pilot study participants allocated to the intervention arm will be invited to participate in an individual semi-structured interview shortly after the end of the program. Interviews will cover potential changes in fertility-related distress and/or sexuality problems experienced by participants, and explore mechanisms that may have brought about such changes. Additionally, experiences of participation, including usage and perceptions of various intervention components will be explored. Interviews will be conducted either in person or via telephone or Zoom, transcribed verbatim and analyzed using reflexive qualitative thematic analysis as described by Braun and Clarke [77].

### Sample size for the full trial

To ensure adequate power (80%) to detect statistically significant differences for each of the two primary outcomes (RCAC and SexFS v2.0 BSP), we estimate a total sample size of 201 participants, assuming a medium effect size (0.5) and α = 0.05. Based on prior work from our research group we anticipate that among these 201 participants, approximately 75 will experience fertility-related distress, 75 will have problems related to their sex lives, and 51 will experience challenges in both areas. The sample size for each primary outcome will be 126 as the 51 experiencing problems in both areas will be included in analysis of both outcomes. To account for an expected attrition rate of 25%, we plan to recruit a total of 252 participants.

### Recruitment

Participants will be recruited through adverts on social media, through patient advocacy organizations, and through oncology and hematology clinics. Individuals interested in participation will contact the research group via the U-CARE platform, phone or email to receive more information about the study (written and oral) and to be assessed for eligibility. Eligibility criteria will be assessed through individual interviews with a research team member. Participants fulfilling eligibility criteria will receive log in details to the U-CARE platform where they will be requested to provide online written informed consent and to respond to the baseline questionnaire (T0). Upon providing consent and returning the questionnaire, participants are included in the study and will subsequently be randomized to either the intervention or control condition.

### Randomization

Participants will be randomized to either intervention or control group using stratified block randomization stratified by sex, and with an allocation ratio of 1:1. A computer-generated randomization sequence will be created through the U-CARE platform and will be concealed for the researchers. Participants will be informed about their assigned group allocation via text message and e-mail. Participants randomized to the intervention group will be invited to the mandatory start-up session, conducted via zoom or telephone, during which a personal set of modules will be recommended. Following this interview and determination of modules, participants will receive instructions on how to access the intervention. Participants randomized to the control condition receive standard care and follow-up.

#### Blinding (masking).

Due to the nature of the intervention, researchers involved in delivering and monitoring the intervention will not be blinded to group allocation of participants.

### Statistical analyses

Sociodemographic and clinical data will be presented as frequencies and percentages (categorical variables), and mean and SD or median and range. Statistical analyses of the full RCT will primarily investigate differences between IG and CG in the primary outcomes (RCAC and PROMIS® SexFS BSP) directly after end of the intervention (T1) taking baseline values into account, using repeated-measures analysis of variance (ANOVA) and/or or linear mixed models (LMM). The main analyses will be performed according to the intention-to-treat principle. Patterns of missing data will be examined and multiple imputation will be used on the bases of the nature of the missing data. Depending on group size, subgroup analyses will be performed for sex, and to assess the potential effects of level of activity in the intervention.

Mediation analysis will be conducted to assess whether effects of the intervention are mediated by the basic psychological needs of SDT. To examine mediation, bootstrapping methods, using the PROCESS tool in SPSS, will be used to estimate and test the indirect effects of the treatment on intervention outcomes directly at end of the intervention (T1) via the basic psychological needs as assessed half-way through the 12-week intervention.

There will be no interim data monitoring since data are self-reported, and as IG participants are followed continuously through the intervention program. All analyses will be conducted in collaboration with external statisticians, uninformed about study participants’ group allocation. Analyses will primarily be conducted in SPSS Statistics version 28 [78] and R version 4.2.2 [79].

### Adverse effects

Adverse effects will be assessed by number of participants reporting possible worsening of symptoms in primary or secondary outcomes. The post-intervention survey further includes a question about potential experiences of worsening of symptoms. Additionally, the exit session will include opportunity to discuss potential adverse effects, and interviews conducted with intervention group participants following the internal pilot study will include a question about potential adverse effects experienced. Finally, throughout the intervention period, participants will be able to contact the research group with any concerns, and the discussion forum will be monitored for posts that may require further assistance.

### Ethics and dissemination

#### Research ethics approval.

Ethical approval for the study procedures has been obtained by the Swedish Ethical Review Authority (Dnr: 2023-02745-01) (S4 Research protocol approved by Ethical Review Authority in S4 File). The present study protocol involved no data collection. For participants of the planned studies presented in the protocol, online written informed consent will be collected (S5 Model consent form in S5 File).

#### Confidentiality.

Participants will be assigned a random identification code through the U-CARE platform. The code key will be stored on a server separate from the collected data, and only members of the research team will have access to the key. All participants will be assigned a pseudonym which will be visible to other participants and moderators when writing a post and commenting in the discussion forum. Researches will be able to connect the pseudonyms to the code number at the stage of analysis. All data will be handled according to the EU General Data Protection Regulation (GDPR).

#### Dissemination policy.

Findings of this trial will be disseminated in the scientific-, clinical- and general and patient communities via presentations at patient organizations, at conferences and through publications in peer-reviewed journals. Additionally, results of the study will be disseminated using social media, including LinkedIn and Facebook. Important modifications to the protocol will be updated in the ISRCTN registry.

## Discussion

The Fex-Can 2.0 intervention is designed to provide young adults with psychoeducational- and behavior change content to alleviate fertility-related distress and sexual problems following cancer. Results of the internal pilot study will demonstrate whether the intervention and study procedures are feasible and acceptable. Results of the RCT will demonstrate whether the Fex-Can 2.0 intervention can reduce fertility-related distress and sexual dysfunction among young adults following cancer.

The present study has numerous strengths to be considered. First, a main strength is the RCT design, allowing for conclusions about the efficacy of the intervention. Second, conducting an internal pilot study prior to the full-scale RCT allows for trialing of key uncertainties, and provides opportunity to make amendments if needed [49]. If progression criteria are met to a satisfactory degree, and the study subsequently proceeds into the full-scale RCT, results from the internal pilot will be included in the main trial, limiting research waste [49]. Third, the adaption and refinement of the original Fex-Can intervention was done in collaboration with patient research partners. Fourth, as recommended by the MRC framework [80], the intervention is grounded in a relevant theory [39] and is thus designed to support participants basic psychological needs as described by SDT. Further, a measure assessing need satisfaction as a source of motivation is included [73,74]. This allows for exploration of whether the intervention facilitates support of the psychological needs, and whether support of the needs is associated with potential changes in primary outcomes. Potential weaknesses should also be mentioned. High attrition rates and low adherence are common in internet-delivered interventions [81]. While this will be evaluated in the internal pilot phase, and several strategies to enhance adherence will be applied (e.g., text message and email reminders, individualized feedback), it is possible that the trial may be susceptible to low rates of adherence. Similarly, there is a risk of low recruitment rates. Previous studies have reported difficulties recruiting participants to interventions targeting sexual and reproductive health [33,82,83]. In the first version of the Fex-Can intervention, recruitment rates were acceptable. However, a proportion of participants did not experience significant problems related to fertility or sexuality and may thus not have been in direct need for support. As such, the internal pilot study will include progression criteria to assess both the feasibility of recruiting a large enough sample for the full-scale trial, as well as the feasibility of recruiting participants with significant fertility-related distress and sexual problems.

In conclusion, previous research has continuously demonstrated the negative effects of cancer and its treatment on fertility and sexuality among young adults, and evidence-based interventions treat these problems are warranted. If proven efficacious, the Fex-Can 2.0 intervention may have significant clinical implications through improving the care provided to young adults diagnosed with cancer.

## Supporting information

S1 File SPIRIT checklist. (DOCX)

S2 File TIDieR checklist. (DOCX)

S3 Table WHO trial registration data. (DOCX)

S4 File Research protocol approved by Swedish Ethical Review Authority. (DOCX)

S5 File Model consent form. (DOCX)
