# Supplementary material for: An internet-delivered psychoeducational intervention (Fex-Can 2.0) targeting fertility-related distress and sexual dysfunction in young adults diagnosed with cancer: Study protocol of a randomized controlled trial with an internal pilot phase
Source: PLoS One. 2025 Apr 29;20(4):e0322368. doi: 10.1371/journal.pone.0322368 (PMC12040239; doi:10.1371/journal.pone.0322368)
Supplement: S3 Table — (DOCX) [file pone.0322368.s003.docx]

| **World Health Organization Trial Registration Data Set** | | |
| --- | --- | --- |
| **Data category** |  | **Information** |
| Primary registry and trial identifying number |  | ISRCTN, ISRCTN18040643 |
| Date of registration in primary registry |  | 12^th^ December 2024 |
| Secondary identifying numbers |  | N/A |
| Source(s) of monetary or material support |  | Swedish Research Council, Forte, Swedish Cancer Society, Cancer Research Funds of Radiumhemmet |
| Primary sponsor(s) |  | Department of Public Health and Caring Sciences, Uppsala University |
| Secondary sponsor(s) |  | N/A |
| Contact for public queries |  | [fexcan@uu.se](mailto:fexcan@uu.se) |
| Contact for scientific queries |  | [lena.wettergren@uu.se](mailto:lena.wettergren@uu.se), [claudia.lampic@umu.se](mailto:claudia.lampic@umu.se) |
| Public title |  | Internet-delivered treatment for fertility distress and sex problems following cancer |
| Scientific title |  | An internet-delivered psychoeducational intervention (Fex-Can 2.0) targeting fertility-related distress and sexual dysfunction in young adults diagnosed with cancer: study protocol of a randomized controlled trial with an internal pilot phase |
| Countries of recruitment |  | Sweden |
| Health condition(s) or problem(s) studies |  | Fertility-related distress and sexual dysfunction following cancer |
| Intervention(s) |  | Intervention group: access to Fex-Can 2.0, an internet-delivered guided self-help intervention. Fex-Can 2.0 is delivered over 12 weeks and consists of modules with educational and behavior change content (e.g., multimedia, interactive components, discussion forum).  Control group: standard care and follow-up. |
| Key inclusion and exclusion criteria |  | Eligibility criteria: diagnosed with cancer within the past 5 years, 18-39 years at study entry, experiencing significant fertility-related distress and/or sexual problems, and preparedness to spend at less 30 minutes per week on the program. Exclusion criteria: inability to communicate in Swedish, suicidality or significant psychiatric condition. |
| Study type |  | Internal pilot study and randomized controlled trial |
| Date of first enrolment |  | April 2025 |
| Target sample size |  | 252 participants |
| Recruitment status |  | Not yet started |
| Primary outcome(s) |  | Fertility-related distress, assessed using the Reproductive Concerns After Cancer scale (RCAC) and sexual function and satisfaction, assessed using the PROMIS® SexFS v2.0 Brief Sexual Profile (SexFS v2.0 BSP). |
| Key secondary outcome(s) |  | Body image (BIS), emotional distress (HADS), health-related quality of life (EORTC QLQ-C30), fertility- and sex-related knowledge, self-efficacy related to fertility and sex life, need satisfaction and frustration (NSFS). |
