# Supplementary material for: An internet-delivered psychoeducational intervention (Fex-Can 2.0) targeting fertility-related distress and sexual dysfunction in young adults diagnosed with cancer: Study protocol of a randomized controlled trial with an internal pilot phase
Source: PLoS One. 2025 Apr 29;20(4):e0322368. doi: 10.1371/journal.pone.0322368 (PMC12040239; doi:10.1371/journal.pone.0322368)
Supplement: S4 File — (DOCX) [file pone.0322368.s004.docx]

**Internet-based treatment of fertility-related distress and sexual problems after cancer – a randomized controlled trial of the Fex-Can 2.0 program**

**PURPOSE AND AIMS**
The aim of the present project is to test the effect of an internet-delivered psychoeducational intervention aiming to reduce fertility concerns and sexual problems in young adults following cancer. As a first step, an internal randomized controlled pilot trial will be conducted. The aim of the pilot trial is to test the Fex-Can (2.0) intervention in young adults in preparation for the full-scale RCT.

**STATE OF THE ART**
Cancer affects large groups of young adults. In Sweden approximately 2000 adults up to age 39 are diagnosed yearly with different types of cancer (1). Both the cancer and its treatment may negatively impact on fertile ability and sex life in both men and women, problems that may interfere with important life goals such as finding a partner and building a family.

*Fertility*
Several cancer treatments have the potential to partially or totally impair patients’ future fertility due to gonadotoxic effects of chemotherapy, radiation of reproductive organs or surgical removal of reproductive tissue (2). Cancer survivors are less likely to ever have biological children than controls without cancer (3). Infertility is associated with psychological distress among cancer survivors and impaired fertility may represent a significant compromise of the quality of survivorship (4).

Female survivors of cancer in reproductive ages report high levels of fertility distress (5). They commonly worry that a pregnancy may increase the risk of a relapse, health risks for offspring, heredity and premature menopause (6). Importantly, reproductive concerns among female patients are associated to long-term depressive symptoms (7), while research on men’s reproductive concerns following cancer is limited. There are few reports of interventions focusing these issues, but a web-based program providing reproductive health and fertility education was found to increase fertility-related knowledge as well as to improve mood and functioning among young breast cancer survivors (8).

*Sexuality and intimacy*
Cancer during young adulthood may lead to negative consequences on sexuality and intimate relationships caused by a combination of physiological changes induced by cancer and its treatment (9). Late effects include unfavorable changes in self-esteem and body image as well as physical complications and symptoms affecting the ability to perform sexual activities (10). Estimates of sexual dysfunction in this group vary by cancer type but are reported by up to 50% of men (11) and women (12,13). A few web-based interventions aiming to increase sexual function and intimacy following cancer treatment have been positively reported for both sexes (14–18). However, few interventions include young patients.

*Patients’ needs of information about fertility and sexual issues*

International and national guidelines recommend that healthcare providers initiate discussions about the impact of cancer treatment on fertility as early as possible; all patients are also to receive information addressing sexual health and dysfunction. Findings have repeatedly shown that young adults want healthcare professionals to raise issues of fertility and sexuality and to provide them with information and advice, but such care is often insufficient or lacking (19,20). Physicians and nurses in cancer care typically perceive a number of barriers that prevent them from initiating conversations about fertility and sexuality matters with their patients (21,22).

**PRELIMINARY AND PREVIOUS RESULTS**

The Fertility and sexuality following cancer (Fex-Can) project was initiated in 2013 and includes a population-based cohort study (23) with an embedded RCT (24). The first version of the intervention (Fex-Can 1.0) showed to be efficient to some extent, but did not reach the hypothesized statistical effects. Process evaluation of that trial has informed the planned changes aimed to optimize the next generation’s intervention (2.0).


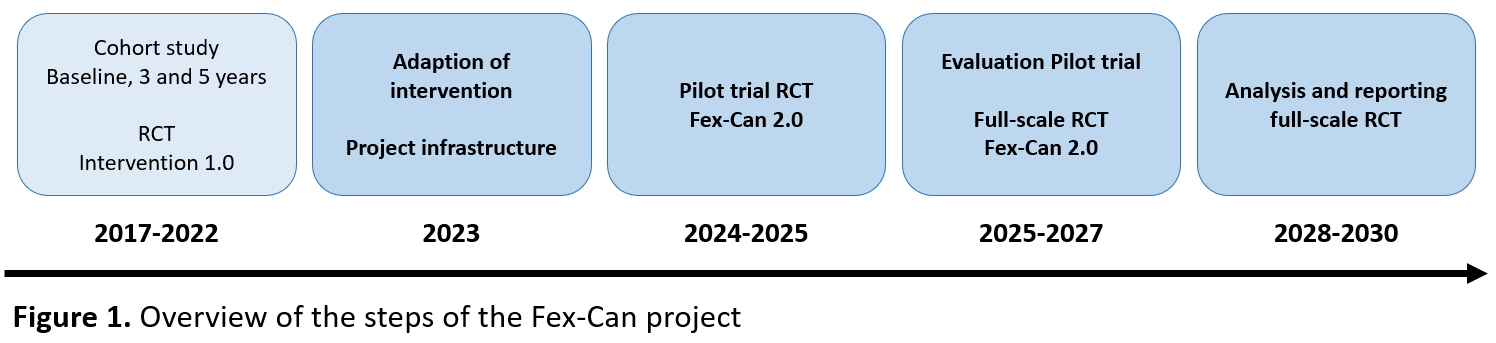


*Fex-Can Cohort study*

The Fex-Can Cohort study includes a national sample of young women and men assessed during the first five years after being diagnosed with cancer. The study combines registry and survey data of young adults with cancer (18-39 years old at diagnosis), and includes a comparison group in similar ages drawn from the general population (N=809). A total of 1010 cancer patients were included at baseline (67% response rate), and the 3-year follow-up was completed with 720 participants (74% response rate following exclusion of 35 individuals due to death or lack of address) and the 5-year follow-up was finalized 2022 (response rate 70%). Potential participants were identified in national quality registries and include breast cancer (women only), cervical cancer, ovarian cancer, lymphoma, testicular cancer and brain tumors.

Sexual dysfunction - prevalence and risk factors

At baseline, 60% of the women with cancer (all diagnoses) reported sexual dysfunction in at least one of the measured domains (25). The women with cancer had significantly more problems, in seven of eight measured domains, than did women of a comparison group in similar ages. Women with gynecological or breast cancer, and those receiving more intensive treatment, were at particular high risk of sexual dysfunction (≥2 domains). Preliminary results of the 3-years follow-up show no improvement in sexual dysfunction and underscore the need for interventions and support. About a third of the men with cancer reported sex problems and they rated erectile dysfunction and lack of interest in having sex to a statistically significantly larger extent than men of the general population.

Fertility-related distress - prevalence and risk factors

At baseline, 54% of the women with cancer and 27% of the men reported fertility distress in at least one dimension, most often concerning their fertile ability and the genetic cancer risk of future children. Patients who had a current child wish and had concurrent symptoms of anxiety or depression were at particular high risk of fertility distress (26).

Received information about treatment impact on fertility and sex life

A majority of both men and women (≈80%) reported that they had received information about the potential impact of their disease/treatment on fertility (27). This indicates a more equal provision of fertility-related information in Sweden than reported in our previous population-based study of young adults diagnosed in 2003-2007 (28). In comparison, only 58% of patients had received information about possible negative impact on their sex life (29), calling for improvement. Importantly, women and men diagnosed with brain tumors stood out at being particular risk of being uninformed about potential future fertility and sexual problems.

*Fex-Can RCT (Intervention 1.0)*

Fex-Can intervention: development and design

The internet-based psycho-educational Fex-Can intervention consists of two programs, Fex-Can Fertility and Fex-Can Sex, aimed to alleviate fertility-related distress and sex problems after cancer, respectively. The programs were delivered over 12 weeks and organized in six successive modules with informational material, texts and exercises. The design, contents and mode of delivery was conceived to facilitate satisfaction of participants’ basic needs according to self-determination theory (30). It was assumed such theoretical orientation would enhance positive health outcomes such as self-efficacy and health-related quality of life (31). Nuanced information and reliable facts were intended to leverage participants’ *competence*. Written and filmed survivor stories as well as interactive quizzes and a discussion forum were included with the goal of helping participants find strategies to handle their concerns/problems by strengthening *autonomy and relatedness*. The development, design, contents and structure of the intervention have been described in detail in previous publications (32–34).

The Fex-Can intervention was developed in a co-creative collaboration with a group of ten former cancer patient and two significant others, as described by (32). This collaboration was initiated in 2014 and is still ongoing with half-day meetings every semester.

Feasibility and efficacy of the Fex-Can 1.0
In 2016 we performed two trials to test the feasibility of delivering the Intervention and the acceptability to patients (33). Based on quantitative data (e.g. evaluation of each module, and log data) and individual interviews with participants, the intervention was found acceptable and feasible.

Subsequently (2017-2018), the Intervention 1.0 was tested in a full RCT with 267 patients (see study protocol (24)). The Fex-Can Fertility program had small to moderate positive effects on fertility-related distress and knowledge (35). The intervention group rated significantly lower distress regarding impact on Child’s health and they also reported better cancer-related fertility knowledge, compared to the control group. Corresponding analyses of the Fex-Can Sexuality program are ongoing.

In conclusion, short-term effects were seen on some of the outcomes, however not in line with what was hypothesized. Still, 60% of participants of the Fex-Can Fertility reported a self-perceived improvement of their problems directly after the intervention (35). Interviews performed with participants post-intervention revealed an overall appreciation with the structure and content of both programs, but suggestions for improvement were also expressed (35). We also observed that uptake of the intervention was low, for example 78% of the RCT participants (Fex-Can Fertility) spent less than 20 minutes in the program during the course of 12 weeks (35).

**PROJECT DESCRIPTION**

*Theory*

The concepts, theories and theoretical models that the project is based on have been developed within care sciences, sociology and psychology. For example, our web-based intervention, is founded on the theory for enhancing and understanding e-health interventions developed by Pingree et al. (2010) aiming to support participants´ self-efficacy by improved competence (feeling able and knowledgeable), autonomy (feeling in control of one’s decisions) and relatedness (connectedness to others) (31). According to self-determination theory (30), if these three basic needs are nurtured they promote motivation and wellbeing which is the basis for the intervention (36). Another cornerstone of our research is the patient involvement, regarded as an integral part of good scientific practice, increasing the quality and relevance of research (37). The adaption of the intervention to 2.0 will involve patient research partners, using the same procedures as practiced when the 1.0 was developed (32).

*Aim*

The aim of the present project is to test the effect of an internet-delivered psychoeducational intervention aiming to reduce fertility concerns and sexual problems in young adults following cancer. As a first step, an internal randomized controlled pilot trial will be conducted. The aim of the pilot trial is to test the Fex-Can (2.0) intervention in young adults in preparation for the full-scale RCT.

*Study design*

A two-armed parallel-group pre-post and follow-up superiority randomized controlled design with a 1:1 allocation ratio. Participants will be randomized to either receive the internet intervention (IG) or to an active control group receiving standard care and access to a website providing links and information about cancer during young adulthood, with focus on fertility and sexuality (CG).

The pilot study will be conducted in a two-armed internal pilot study, following the same procedures as described above. An internal pilot study uses the outcomes that are planned for the main study and the results can, if the pilot trial reaches the set targets, be included in the full-scale trial. The study aims to investigate certain elements of the trial which will be assessed against pre-specified criteria (38).

*Participants and eligibility criteria*

Study participants will be eligible if 1) diagnosed with cancer (≤5 years) during young adulthood (18-39 years); 2) self-report fertility distress and/or sexual dysfunction; 3) troubled by their distress/problems; 4) prepared and agree to work in the intervention program for at least 30 minutes per week; and 5) are able to communicate in Swedish. Participants will be recruited through social media (e.g. Instagram, Facebook), oncology and hematology clinics in Uppsala, Umeå and Stockholm, and patient organizations such as Young Cancer (Ung Cancer) and the Swedish Breast Cancer Association.

To detect a statistically significant difference with regards to fertility-related distress (using the Reproductive Concerns After Cancer scale) and/or sexual problems (using the Satisfaction with sex life domain of the SexFS v.2) with a power of 80%, medium effect size (0.5) and α = 0.05 a sample size of 126 completers with fertility distress and 126 completers with sexual dysfunction will be needed (at the 3-months follow-up assessment). In our previous intervention, the Fex-Can 1.0, 40% of the participants had problems in both areas and will therefore possible to evaluate effects on fertility distress and sexual dysfunction. Therefore, we will recruit 75 experiencing fertility-related distress, 75 experiencing sex problems, and additionally 51 who experience problems in both areas. We estimate an attrition rate of 25%, thus we will recruit a total of 252 participants.

The first 70 participants for the full-scale RCT will be recruited for the internal pilot study; 35 in the intervention group and 35 controls. For the interviews conducted after the intervention we aim to include all intervention group participants (i.e., 35 participants).

*Randomization*
Consenting individuals will be randomized to either intervention (IG) or control group (CG). This will be performed by an external statistician not involved in the data collection process, by stratified block randomization taking account of sex.

*Intervention 2.0*The next generation of the intervention (2.0) aims to overcome the shortcomings identified with the first intervention (1.0). This includes that some participants didn’t appear to have an evident need of support, and overall low adherence resulting in a low dose of the given treatment. Based on this we will modify the intervention as presented below (Table 1).

**Table 1.** Descriptions of the shortcomings of the first intervention and the proposed changes

| ***Shortcoming*** | ***Proposed changes*** | ***Intervention 2.0*** |
| --- | --- | --- |
| Some participants not in apparent need of support | Increase thresholds for inclusion criteria | Participants troubled by fertility-related distress (defined as >4 in at least two domains of the RCAC) and/or sex problems (defined as 1 SD above the general population in the SexFS v.2.0) |
| Problems with both fertility and sexuality | Combine the previous two programs into one | Include modules to overcome both fertility-related distress and sexual problems |
| Low activity and adherence | Add new features to increase participants usage (dose) | Sessions with team member (face-to-face, Zoom, telephone) at start and end of program |
|  |  | Recommend specific modules based on participant’s personal situation |
|  |  | Personal written feedback on exercises |

The organization of the Fex-Can program, in successive modules together with a moderated discussion forum, was appreciated, so this structure will remain intact. Each module will target a specific aspect of sexuality or fertility after cancer (see Figure 2) and includes short articles (educational and behavior change content), multimedia (e.g. short videos of persons with fertility concerns and/or sexual problems following cancer), interactive online activities (e.g. self-monitoring exercises), and feedback support (e.g. discussion forum, quiz). The exercises in the program aim at increasing sexual pleasure and functioning, by for example targeting body awareness and acceptance. Other exercises in the program aim at helping users to find new ways of handling their threatened or lost fertility by for example improving problem-solving skills, mindfulness and acceptance.


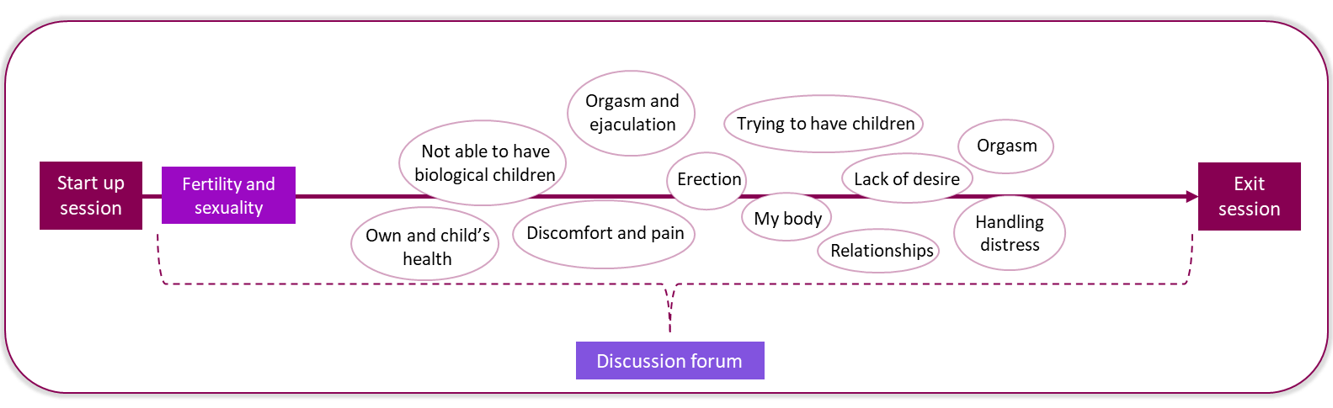


**Figure 2.** Structure of intervention 2.0

Participants’ problems and needs related to fertility and sex will be assessed in a start-up session with a member of the research team (face-to-face, telephone or Zoom). Based on each participant’s expectations and goals of the program, a set of modules will be recommended (see Figure 2). All intervention group participants will also be offered an exit session at the end of the program to assess experiences of the participation and current status of problems.

*Outcomes*

Outcomes will be assessed before the intervention, at the end of intervention, and 3 months after the end of the intervention. The primary outcomes are fertility distress and sexual function.

Fertility distress will be measured with the Reproductive Concerns After Cancer (RCAC) scale assessing emotional distress related to reproductive problems in six domains (e.g. fertility potential, child health and disclosure of fertility status) (39). Responses are recorded on a 5-point scale, with mean scores of >4 indicating distress. The RCAC has satisfactory internal consistency and construct validity (40).

Sexual function will be measured with the domains of the PROMIS Sexual Function and Satisfaction measures (SexFS version 2.0) (41): Satisfaction with sex life, Interest in sexual activity, Orgasm ability and pleasure, Vaginal lubrication, Vaginal discomfort, Vulvar discomfort and Erectile Function. Domain scores are transformed to a T-score metric where 50 represents the mean for sexually active American adults (SD=10) (41). As recommended by the PROMIS Network (http://www.nihpromis.org/), a cut-off of 1 SD from mean is used to define sexual dysfunction. The SexFS has shown adequate content, construct and known-groups validity as well as test-retest reliability (41).

Health-related quality of life will be measured by the EORTC QLQ-C30. The instrument has demonstrated good psychometric properties in cancer populations (42).

Body image disturbance associated with the cancer experience will be assessed with the Body Image Scale (BIS) (43) shown to have clinical validity, test-retest reliability and satisfactory internal consistency.
Anxiety and depression will be assessed using the Hospital Anxiety and Depression scale (HADS) with satisfactory internal consistency and validity (44).
Fertility-related knowledge will be examined by a study-specific questionnaire with 10 items measuring perceived level of knowledge about general and cancer-related fertility issues. Self-efficacy will be examined by a study-specific questionnaire measuring confidence in own ability to handle situations, thoughts and emotions related to the threat of infertility (6 items) and sexual activity (6 items).
Additional characteristics include socio-demographic variables (e.g. education and family situation), reproductive aspects (e.g. desire for children, fertility problems pre/post diagnosis, menopausal symptoms), and sexual aspects (e.g. satisfaction with sex life pre-diagnosis).
Clinical data will be collected through self-reports (cancer diagnosis and treatment).

*Data analyses*

Besides descriptive and inferential statistics, statistical methods used for the full-scale RCT will be linear mixed models, focused on comparing the primary outcomes between intervention and control group after the intervention, as well as changes over time. Intention-to-treat analyses will be applied. Interviews will be analyzed using qualitative content analysis (45).

Feasibility of the pilot trial will be evaluated by assessing the specific procedures described below (see Table 2), in accordance with the CONSORT 2010 statement for randomized pilot and feasibility trials (46). Based on the fulfillment of the predefined progression criteria, a decision will be taken whether to proceed immediately to a full-scale trial, proceed with amendments, or not to proceed (46). Outcomes between groups (intervention / control) will be compared at pre-intervention, post-intervention and at the three months follow-up using Student’s t-test or Mann-Whitney U test, according to distribution of data.

**Table 2**. Progression criteria for evaluation of the pilot trial

| **Outcome** | **Evaluation** | **Progression criterion** |
| --- | --- | --- |
| *Recruitment* | Number identified via Internet, patient organizations, advertising | 50 potential participants/year |
|  | Percentage assessed for eligibility and fulfilling inclusion criteria | Not specified |
|  | Percentage fulfilling inclusion criteria and enrolled (of total number invited) | >60% of those consenting to participate |
|  | Reasons for non-participation | Not specified |
|  | Reasons for ineligibility | Not specified |
| *Attrition* | Rate of trial dropout | <20% |
| *Participants adherence to intervention* | Percentage opening all assigned modules | >50% |
|  | Percentage posting at least one message in the discussion forum | >15% |
|  | Percentage completing at least half of the exercises in assigned modules | >50% |
|  | Percentage spending on average 15 minutes per week in the program | >50% |
|  | Percentage completing sessions (baseline and post-treatment) | >60% |
| *Resources needed to deliver the intervention* | Time for team members to conduct individual sessions (start and exit), give feedback and technical support | No criteria set |
|  | Reminder contacts needed at different time points | No criteria set |

*Procedure*

Participants will be recruited through social media (e.g., Facebook, Instagram), oncology and hematology clinics, and through patient organizations. Interested participants will contact the research group through the U-CARE portal, telephone or e-mail. Written information will then be sent out by post, and an appointment will be set up to deliver oral information about the study and to assess eligibility. Eligible participants will be sent a baseline survey and a written informed consent form. Upon returning the consent form, participants will be randomized to either intervention- or control group by an external statistician. Randomized participants will receive access to their respective condition (i.e., the internet-delivered psychoeducational intervention or the tailored informational website). The intervention will be carried out from the established U-CARE Portal at Uppsala University, an internet-based infrastructure for delivery and evaluation of internet-based interventions.

**CLINICAL SIGNIFICANCE**

The Fex-Can Cohort study has shown that fertility-related distress and sex problems are common among young adults diagnosed with cancer. If the results indicate that the trial is feasible, we will continue with a full-scale RCT (38). Our close collaboration with clinicians and representatives of the national quality registries will facilitate implementation.

**REFERENCES**

1. Miller KD, Fidler-Benaoudia M, Keegan TH, Hipp HS, Jemal A, Siegel RL. Cancer statistics for adolescents and young adults, 2020. CA: A Cancer Journal for Clinicians. 2020;70(6):443–59.

2. Rodriguez-Wallberg KA. Principles of Cancer Treatment: Impact on Reproduction. In: Quinn GP, Vadaparampil ST, editors. Reproductive Health and Cancer in Adolescents and Young Adults [Internet]. Dordrecht: Springer Netherlands; 2012 [cited 2023 Jan 26]. p. 1–8. (Advances in Experimental Medicine and Biology). Available from: https://doi.org/10.1007/978-94-007-2492-1_1

3. Madanat LMS, Malila N, Dyba T, Hakulinen T, Sankila R, Boice JD, et al. Probability of parenthood after early onset cancer: A population-based study. Int J Cancer [Internet]. 2008 Dec 15 [cited 2023 Jan 26];123(12):2891–8. Available from: https://www.ncbi.nlm.nih.gov/pmc/articles/PMC2730156/

4. Duffy C, Allen S. Medical and Psychosocial Aspects of Fertility After Cancer. Cancer J [Internet]. 2009 [cited 2023 Jan 26];15(1):27–33. Available from: https://www.ncbi.nlm.nih.gov/pmc/articles/PMC2719717/

5. Benedict C, Thom B, Friedman MD DN, Pottenger E, Raghunathan N, Kelvin JF. Fertility information needs and concerns post-treatment contribute to lowered quality of life among young adult female cancer survivors. Support Care Cancer [Internet]. 2018 Jul [cited 2022 Nov 24];26(7):2209–15. Available from: https://www.ncbi.nlm.nih.gov/pmc/articles/PMC5984121/

6. Gorman JR, Su HI, Roberts SC, Dominick SA, Malcarne VL. Experiencing reproductive concerns as a female cancer survivor is associated with depression. Cancer [Internet]. 2015 [cited 2022 Nov 4];121(6):935–42. Available from: https://onlinelibrary.wiley.com/doi/abs/10.1002/cncr.29133

7. Gorman JR, Malcarne VL, Roesch SC, Madlensky L, Pierce JP. Depressive Symptoms among Young Breast Cancer Survivors: The Importance of Reproductive Concerns. Breast Cancer Res Treat [Internet]. 2010 Sep [cited 2022 Nov 21];123(2):477–85. Available from: https://www.ncbi.nlm.nih.gov/pmc/articles/PMC2888956/

8. Meneses K, McNees P, Azuero A, Jukkala A. Development of the Fertility and Cancer Project: an Internet approach to help young cancer survivors. Oncol Nurs Forum. 2010 Mar;37(2):191–7.

9. Bober SL, Varela VS. Sexuality in Adult Cancer Survivors: Challenges and Intervention. JCO [Internet]. 2012 Oct 20 [cited 2022 Nov 21];30(30):3712–9. Available from: https://ascopubs.org/doi/full/10.1200/JCO.2012.41.7915

10. Burwell SR, Case LD, Kaelin C, Avis NE. Sexual problems in younger women after breast cancer surgery. J Clin Oncol. 2006 Jun 20;24(18):2815–21.

11. Arden-Close E, Eiser C, Pacey A. Sexual functioning in male survivors of lymphoma: a systematic review (CME). J Sex Med. 2011 Jul;8(7):1833–41.

12. Fobair P, Stewart SL, Chang S, D’Onofrio C, Banks PJ, Bloom JR. Body image and sexual problems in young women with breast cancer. Psychooncology. 2006 Jul;15(7):579–94.

13. Acquati C, Zebrack BJ, Faul AC, Embry L, Aguilar C, Block R, et al. Sexual functioning among young adult cancer patients: A 2-year longitudinal study. Cancer. 2018 Jan 15;124(2):398–405.

14. Brotto LA, Erskine Y, Carey M, Ehlen T, Finlayson S, Heywood M, et al. A brief mindfulness-based cognitive behavioral intervention improves sexual functioning versus wait-list control in women treated for gynecologic cancer. Gynecol Oncol. 2012 May;125(2):320–5.

15. Chambers SK, Occhipinti S, Schover L, Nielsen L, Zajdlewicz L, Clutton S, et al. A randomised controlled trial of a couples-based sexuality intervention for men with localised prostate cancer and their female partners. Psycho-Oncology [Internet]. 2015 [cited 2023 Jan 24];24(7):748–56. Available from: https://onlinelibrary.wiley.com/doi/abs/10.1002/pon.3726

16. Schover LR, Strollo S, Stein K, Fallon E, Smith T. Effectiveness trial of an online self-help intervention for sexual problems after cancer. J Sex Marital Ther. 2020;46(6):576–88.

17. Wootten AC, Pillay B, Abbott JAM. Can sexual outcomes be enhanced after cancer using online technology? Current Opinion in Supportive and Palliative Care [Internet]. 2016 Mar [cited 2022 Sep 29];10(1):81–6. Available from: https://journals.lww.com/co-supportiveandpalliativecare/Fulltext/2016/03000/Can_sexual_outcomes_be_enhanced_after_cancer_using.16.aspx

18. Classen CC, Chivers ML, Urowitz S, Barbera L, Wiljer D, O’Rinn S, et al. Psychosexual distress in women with gynecologic cancer: a feasibility study of an online support group. Psycho-Oncology [Internet]. 2013 [cited 2022 Nov 29];22(4):930–5. Available from: https://onlinelibrary.wiley.com/doi/abs/10.1002/pon.3058

19. Crawshaw MA, Glaser AW, Hale JP, Sloper P. Male and female experiences of having fertility matters raised alongside a cancer diagnosis during the teenage and young adult years. Eur J Cancer Care (Engl). 2009 Jul;18(4):381–90.

20. Daly C, Micic S, Facey M, Speller B, Yee S, Kennedy ED, et al. A review of factors affecting patient fertility preservation discussions & decision-making from the perspectives of patients and providers. European Journal of Cancer Care [Internet]. 2019 [cited 2023 Jan 23];28(1):e12945. Available from: https://onlinelibrary.wiley.com/doi/abs/10.1111/ecc.12945

21. Dyer K, das Nair R. Why Don’t Healthcare Professionals Talk About Sex? A Systematic Review of Recent Qualitative Studies Conducted in the United Kingdom. The Journal of Sexual Medicine [Internet]. 2013 Nov 1 [cited 2023 Jan 3];10(11):2658–70. Available from: https://www.sciencedirect.com/science/article/pii/S1743609515301715

22. Lampic C, Wettergren L. Oncologists’ and pediatric oncologists’ perspectives and challenges for fertility preservation. Acta Obstet Gynecol Scand. 2019 May;98(5):598–603.

23. Wettergren L, Ljungman L, Micaux Obol C, Eriksson LE, Lampic C. Sexual dysfunction and fertility-related distress in young adults with cancer over 5 years following diagnosis: study protocol of the Fex-Can Cohort study. BMC Cancer [Internet]. 2020 Aug 5 [cited 2022 Sep 29];20(1):722. Available from: https://doi.org/10.1186/s12885-020-07175-8

24. Lampic C, Ljungman L, Micaux Obol C, Eriksson LE, Wettergren L. A web-based psycho-educational intervention (Fex-Can) targeting sexual dysfunction and fertility-related distress in young adults with cancer: study protocol of a randomized controlled trial. BMC Cancer [Internet]. 2019 Apr 11 [cited 2022 Sep 29];19(1):344. Available from: https://doi.org/10.1186/s12885-019-5518-3

25. Wettergren L, Eriksson LE, Bergström C, Hedman C, Ahlgren J, Smedby KE, et al. Prevalence and risk factors for sexual dysfunction in young women following a cancer diagnosis – a population-based study. Acta Oncologica [Internet]. 2022 Oct 3 [cited 2022 Nov 21];61(10):1165–72. Available from: https://www.tandfonline.com/doi/full/10.1080/0284186X.2022.2112283

26. Rodriguez-Wallberg KA, Ahlgren J, Smedby KE, Gorman JR, Hellman K, Henriksson R, et al. Prevalence and predictors for fertility-related distress among 1010 young adults 1.5 years following cancer diagnosis – results from the population-based Fex-Can Cohort study. Acta Oncologica [Internet]. 2023 [cited 2023 Nov 6];0(0):1–8. Available from: https://doi.org/10.1080/0284186X.2023.2272291

27. Wide A, Wettergren L, Ahlgren J, Smedby KE, Hellman K, Henriksson R, et al. Fertility-related information received by young women and men with cancer - a population-based survey. Acta Oncol. 2021 Aug;60(8):976–83.

28. Armuand GM, Rodriguez-Wallberg KA, Wettergren L, Ahlgren J, Enblad G, Höglund M, et al. Sex Differences in Fertility-Related Information Received by Young Adult Cancer Survivors. JCO [Internet]. 2012 Jun 10 [cited 2022 Sep 30];30(17):2147–53. Available from: https://ascopubs.org/doi/10.1200/JCO.2011.40.6470

29. Bergström C, Lampic C, Roy R, Hedman C, Ahlgren J, Ståhl O, et al. Do young adults with cancer receive information about treatment-related impact on sex life? Results from a population-based study. Cancer Medicine [Internet]. [cited 2023 Feb 21];n/a(n/a). Available from: https://onlinelibrary.wiley.com/doi/abs/10.1002/cam4.5672

30. Ryan RM, Deci EL. Self-determination theory and the facilitation of intrinsic motivation, social development, and well-being. American Psychologist. 2000;55:68–78.

31. Pingree S, Hawkins R, Baker T, DuBenske L, Roberts LJ, Gustafson DH. The Value of Theory for Enhancing and Understanding e-Health Interventions. Am J Prev Med [Internet]. 2010 Jan [cited 2023 Jan 26];38(1):103–9. Available from: https://www.ncbi.nlm.nih.gov/pmc/articles/PMC2826889/

32. Winterling J, Wiklander M, Obol CM, Lampic C, Eriksson LE, Pelters B, et al. Development of a Self-Help Web-Based Intervention Targeting Young Cancer Patients With Sexual Problems and Fertility Distress in Collaboration With Patient Research Partners. JMIR Res Protoc [Internet]. 2016 Apr 12 [cited 2022 Sep 30];5(2):e60. Available from: http://www.researchprotocols.org/2016/2/e60/

33. Wiklander M, Strandquist J, Obol CM, Eriksson LE, Winterling J, Rodriguez-Wallberg KA, et al. Feasibility of a self-help web-based intervention targeting young cancer patients with sexual problems and fertility distress. Support Care Cancer [Internet]. 2017 Dec [cited 2022 Nov 21];25(12):3675–82. Available from: http://link.springer.com/10.1007/s00520-017-3793-6

34. Micaux C. Web-based support for young adults with reproductive concerns following cancer - development, process and outcome evaluation of a self-help psychoeducational intervention. Karolinska Institutet: Universitetsservice US-AB.; 2021.

35. Micaux C, Wiklander M, Eriksson LE, Wettergren L, Lampic C. Efficacy of a Web-Based Psychoeducational Intervention for Young Adults With Fertility-Related Distress Following Cancer (Fex-Can): Randomized Controlled Trial. JMIR Cancer [Internet]. 2022 Mar 29 [cited 2022 Sep 29];8(1):e33239. Available from: https://cancer.jmir.org/2022/1/e33239

36. Obol CM, Lampic C, Wettergren L, Ljungman L, Eriksson LE. Experiences of a web-based psycho-educational intervention targeting sexual dysfunction and fertility distress in young adults with cancer—A self-determination theory perspective. PLOS ONE [Internet]. 2020 Jul 22 [cited 2022 Sep 30];15(7):e0236180. Available from: https://journals.plos.org/plosone/article?id=10.1371/journal.pone.0236180

37. Oliver S, Liabo K, Stewart R, Rees R. Public involvement in research: making sense of the diversity. J Health Serv Res Policy. 2015 Jan;20(1):45–51.

38. Herbert E, Julious SA, Goodacre S. Progression criteria in trials with an internal pilot: an audit of publicly funded randomised controlled trials. Trials [Internet]. 2019 Aug 9 [cited 2023 Jan 24];20(1):493. Available from: https://doi.org/10.1186/s13063-019-3578-y

39. Gorman JR, Su HI, Pierce JP, Roberts SC, Dominick SA, Malcarne VL. A multidimensional scale to measure the reproductive concerns of young adult female cancer survivors. J Cancer Surviv [Internet]. 2014 Jun [cited 2022 Nov 21];8(2):218–28. Available from: http://link.springer.com/10.1007/s11764-013-0333-3

40. Gorman JR, Pan-Weisz TM, Drizin JH, Su HI, Malcarne VL. Revisiting the Reproductive Concerns After Cancer (RCAC) scale. Psycho-Oncology [Internet]. 2019 [cited 2023 Jan 20];28(7):1544–50. Available from: https://onlinelibrary.wiley.com/doi/abs/10.1002/pon.5130

41. Weinfurt KP, Lin L, Bruner DW, Cyranowski JM, Dombeck CB, Hahn EA, et al. Development and Initial Validation of the PROMIS(®) Sexual Function and Satisfaction Measures Version 2.0. J Sex Med. 2015 Sep;12(9):1961–74.

42. Aaronson NK, Ahmedzai S, Bergman B, Bullinger M, Cull A, Duez NJ, et al. The European Organization for Research and Treatment of Cancer QLQ-C30: A Quality-of-Life Instrument for Use in International Clinical Trials in Oncology. JNCI: Journal of the National Cancer Institute [Internet]. 1993 Mar 3 [cited 2023 Jan 23];85(5):365–76. Available from: https://doi.org/10.1093/jnci/85.5.365

43. Hopwood P, Fletcher I, Lee A, Al Ghazal S. A body image scale for use with cancer patients. European Journal of Cancer [Internet]. 2001 Jan 1 [cited 2022 Nov 21];37(2):189–97. Available from: https://www.sciencedirect.com/science/article/pii/S0959804900003531

44. Bjelland I, Dahl AA, Haug TT, Neckelmann D. The validity of the Hospital Anxiety and Depression Scale: An updated literature review. Journal of Psychosomatic Research [Internet]. 2002 Feb 1 [cited 2023 Jan 23];52(2):69–77. Available from: https://www.sciencedirect.com/science/article/pii/S0022399901002963

45. Graneheim UH, Lundman B. Qualitative content analysis in nursing research: concepts, procedures and measures to achieve trustworthiness. Nurse Education Today [Internet]. 2004 Feb [cited 2023 Feb 24];24(2):105–12. Available from: https://linkinghub.elsevier.com/retrieve/pii/S0260691703001515

46. Eldridge SM, Chan CL, Campbell MJ, Bond CM, Hopewell S, Thabane L, et al. CONSORT 2010 statement: extension to randomised pilot and feasibility trials. BMJ [Internet]. 2016 Oct 24 [cited 2023 Jan 27];355:i5239. Available from: https://www.bmj.com/content/355/bmj.i5239
