# Supplementary material for: An internet-delivered psychoeducational intervention (Fex-Can 2.0) targeting fertility-related distress and sexual dysfunction in young adults diagnosed with cancer: Study protocol of a randomized controlled trial with an internal pilot phase
Source: PLoS One. 2025 Apr 29;20(4):e0322368. doi: 10.1371/journal.pone.0322368 (PMC12040239; doi:10.1371/journal.pone.0322368)
Supplement: S5 File — (DOCX) [file pone.0322368.s005.docx]

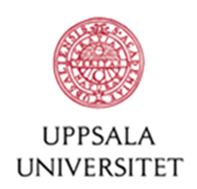

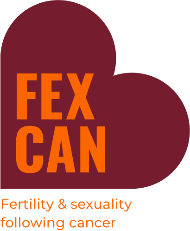


**Internet-delivered treatment for fertility-related distress and sexual problems following cancer**

**Consent to participate in the study**

I have received written information about the study and I have had opportunity to pose questions. I get to keep the written information.

- I consent to participate in the project *Internet-delivered treatment for fertility-related distress and sexual problems following cancer.*
- I consent to that those responsible for the project get to use my personal information for purposes that are stated in the written information.

| Place and date | Signature |
| --- | --- |
|  |  |
|  | Print name |
|  |  |

***We need you phone number and e-mail address!***
We will text/e-mail you with log in details for the internet-delivered program. Please provide your phone number and e-mail address below.

Phone number:

E-mail address:
